# Successful treatment of iodixanol-induced type II Kounis syndrome with an intra-aortic balloon pump: a case report and literature review

**DOI:** 10.3389/fcvm.2025.1652423

**Published:** 2025-10-09

**Authors:** Naiju Zhang, Junran Gao, Zhenjie Wang, Tianping Chen

**Affiliations:** ^1^ Department of Pharmacy, First Affiliated Hospital of Bengbu Medical University, Anhui Engineering Technology Research Center of Biochemical Pharmaceutical, Institute of Emergency and Critical Care Medicine, Branch of National Clinical Research Center for Infectious Diseases, Bengbu, China; ^2^ Department of Emergency Surgery, Institute of Emergency and Critical Care Medicine, First Affiliated Hospital of Bengbu Medical University, Bengbu, China; ^3^ Department of Cardiology, First Affiliated Hospital of Bengbu Medical University, Bengbu, Anhui, China

**Keywords:** iodixanol, contrast media, Kounis syndrome, intra-aortic balloon pump, case report, literature review

## Abstract

**Background:**

Kounis syndrome (KS) is an acute coronary syndrome triggered by hypersensitivity reactions and is often underrecognized in clinical practice. This report describes a case of iodixanol-induced KS that was refractory to standard emergency medications but successfully managed with an intra-aortic balloon pump (IABP), along with a review of similar published cases.

**Case summary:**

A 77-year-old woman with a history of chest pain underwent coronary angiography with iodixanol. During the procedure, she developed progressive bradycardia, hypotension, and convulsions. Despite immediate cardiopulmonary resuscitation and the administration of emergency medications, hypotension persisted. Hemodynamic stability was rapidly achieved following IABP implantation, with complete resolution of symptoms and no complications. This case represents the first reported instance of iodixanol-induced KS that was unresponsive to conventional vasoactive drugs and required IABP for stabilization. A review of the literature identified four prior cases of iodixanol-induced KS, all of which were successfully managed with medical therapy alone, highlighting the unique severity and therapeutic challenge of the present case.

**Conclusion:**

This is the first reported use of IABP for iodixanol-induced KS, suggesting its potential role in refractory cases.

## Introduction

Iodixanol is the only Food and Drug Administration-approved iso-osmolar, nonionic, dimeric hydrophilic contrast agent in the United States and is more viscous than monomeric agents. It is also the only contrast medium approved for use in cardiac computed tomographic angiography to aid in diagnosing suspected coronary artery disease (1). Studies have shown that iodixanol use in coronary angiography is associated with low heart rate variability, fewer and milder side effects, and favorable prognostic outcomes—even in patients with chronic kidney disease or diabetes mellitus (DM) (2).

In contrast, Kounis syndrome (KS), or allergic acute coronary syndrome, is a potentially life-threatening condition characterized by acute coronary events—such as coronary artery spasm, myocardial infarction, or stent thrombosis—triggered by hypersensitivity reactions (3). KS patients present with signs and symptoms of acute coronary syndrome while also showing evidence of an acute hypersensitivity reaction (3). These reactions, which can be induced by medications (including contrast agents), environmental exposures, foods, or coronary stents, involve the release of allergic mediators such as histamine, leading to coronary vasospasm and myocardial ischemia (4). First described in 1991, KS links allergic stimuli with clinical manifestations of chest pain and electrocardiographic (ECG) changes indicative of ischemia (5). The underlying mechanism involves the activation of mast cells, platelets, and inflammatory cells such as macrophages and T lymphocytes, culminating in a combined allergic and ischemic event (6).

Here, we report a rare case of KS type II caused by the contrast agent iodixanol in a patient with two risk factors, namely, hypertension and diabetes. KS rapidly reversed after iodixanol was withdrawn, and cardiopulmonary resuscitation (CPR), emergency drugs and an intra-aortic balloon pump (IABP) were used. Informed consent was obtained from the patient for publication of this case report.

## Case presentation

**Figure 1:**
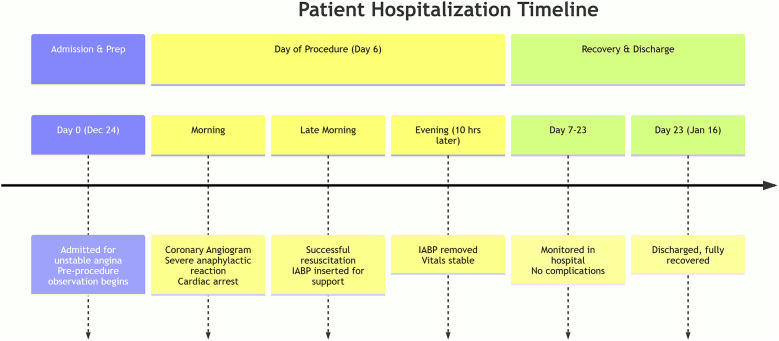
A detailed timeline of the patient's clinical course from admission to hospital discharge.

A 77-year-old Chinese woman (body mass index of 28.4 kg/m^2^) was admitted to the Department of Cardiology at the First Affiliated Hospital of Bengbu Medical University in Bengbu, China, on December 24, 2021, due to a two-month history of paroxysmal chest pain. Three days earlier, that is, December 21, 2021, local hospital color Doppler echocardiography suggested a small amount of pulmonary and mitral regurgitation and reduced left ventricular diastolic function. Chest computed tomography revealed little inflammation in both lungs and increased heart shadow, coronary artery and great vessel sclerosis. Two days earlier, coronary angiography performed with the contrast agent iohexol 50 ml revealed left main trunk tail stenosis 90%, anterior descending branch opening stenosis 50%, proximal segment stenosis 90%, middle segment stenosis 90%, circumflex branch opening stenosis 40%, proximal right crown stenosis 50%, posterior trigeminal anterior stenosis 90%, posterior descending branch opening stenosis 80%, and left ventricular posterior branch opening stenosis 80% on December 22, 2021. Without improvement in treatment at the local hospital, coronary angiography is recommended for a definite diagnosis. Currently, the patient is admitted to our department for “coronary heart disease (CHD), unstable angina pectoris” in the outpatient department of our hospital for further diagnosis and treatment (Figure 1). The patient's medical history included a 20-year history of hypertension that was well controlled with the oral antihypertensive drug irbesartan 150 mg once a day regularly and a three-year history of type 2 DM that was well controlled with the oral hypoglycemic drug glimepiride 2 mg twice a day regularly. He had a history of “appendicitis surgery”. The patient did not have a history of food or drug allergies. No family history of allergic or coronary disease was reported. The patient was awake and alert when she was admitted to the hospital; her temperature was 36.5 °C, her heart rate was 78 beats/min, her respiratory rate was 18 breaths/min, and her blood pressure was 163/74 mmHg. Six days after admission, coronary angiography was performed in the catheterization room at 08:15 on December 30, 2021. The patient was placed in a supine position, and conventional disinfection techniques were applied. The left radial artery was successfully punctured via the modified Seldinger method with 1% lidocaine for local anesthesia. A 6F vascular sheath was inserted, followed by the introduction of JL3.5 and JR3.5 angiography catheters through the sheath. Left coronary angiography was then conducted using iodixanol as the contrast agent.

The results revealed approximately 85% stenosis at the tail of the left main trunk, involving the anterior descending branch and the opening of the circumflex branch; approximately 85%–90% stenosis at the proximal part of the left anterior descending branch; approximately 85%–90% stenosis at the proximal part of the first diagonal branch; and approximately 80% stenosis at the distal part. The forward flow was TIMI 3. Approximately 60%–85% of the left circumflex branch is narrowed in the proximal middle segment, with TIMI 3 flowing forward. After the JL3.5 angiography catheter was inserted into the JR3.5 angiography catheter, the patient initially experienced an increase in blood pressure to 215/85 mmHg, a heart rate of 90 beats per min, and 23 breaths per min. Twenty-three minutes later, a progressive decrease in heart rate (the minimum is 38 beats/min) and blood pressure (the minimum is 60/40 mmHg) occurred, along with unconsciousness and convulsive symptoms. Angiography revealed severe spasm and stenosis of the left coronary artery. CPR was immediately performed, and 5 mg of nitroglycerine was repeatedly administered to the left coronary artery, while 0.5 mg of atropine, 2 mg of norepinephrine, 1 mg of epinephrine, 10 mg of dexamethasone, and 1 g of sodium bicarbonate were immediately administered multiple times for rescue. The patient's consciousness became clear, and her heart rate was maintained at 132 beats per min, but her blood pressure was still low (100/55 mmHg), and her oxygen saturation was 93%. Immediate puncture of the right femoral artery and implantation of an IABP balloon counterpulsation device with an assist ratio of 1:1 as an adjunctive circulatory device were performed. Following IABP implantation, the circulatory status dramatically stabilized, with blood pressure (107/60 mmHg) and heart rate (91 beats per min) remaining within the normal range and an oxygen saturation of 99%.

The counterpulsation pressure fluctuates between 142 and 168 mmHg. Bedside echocardiography revealed no pericardial effusion, and the ejection fraction was approximately 50%. The patient's symptoms resolved within the same period.

After approximately 30 min of observation, the vital signs were stable, and the level of consciousness was clear. At the end of the operation, the left radial artery sheath was removed, and the radial artery was wrapped with sterile dressings. No obstruction was observed, and the patient was diagnosed with vasospasm caused by an allergic reaction to the contrast agent (KS type II) and subsequently transferred to the coronary care unit (CCU) for observation. A total of 3000 U of heparin and 50 ml of iodixanol contrast agent were used during the operation, and the samples were returned to the CCU at 10:00 for observation.

Ten hours later, the patient's vital signs were stable with no complaints of discomfort; her blood pressure was 130/70 mmHg, her heart rate was 85 beats/min, and her blood oxygen saturation was 100%. Therefore, IABP assistance was removed. No palpitations, chest tightness, sweating, dizziness or other discomfort was found during the extraction process, and there were no IABP-related complications, such as bleeding or infection. Seventeen days later, she was discharged with good recovery. The patient had no significant long-term sequelae at the clinical follow-up visit three years later.

The patient provided this perspective: “I have CHD, and my local hospital was unable to provide the necessary treatment. As a result, I transferred to your hospital. During the coronary angiography procedure, I experienced anaphylactic shock, which posed a life-threatening risk. Fortunately, you intervened promptly and saved my life. I am deeply grateful for your medical expertise and skills. Later, we went to another large hospital for coronary artery bypass surgery without iodine contrast, and the operation process went smoothly. Now everything is fine! ”

Informed consent was obtained from the patient for publication of these case report details.

## Literature review

Generally, an allergic reaction to iodixanol is rare, but sometimes it can be life-threatening (7). We performed a literature review to determine whether other patients had experienced iodixanol-associated KS. Literature searches written in English and Chinese were conducted through PubMed and the China National Knowledge Internet from the establishment of the database to May 12, 2025. Finally, three articles (with a total of three cases) reporting iodixanol-associated KS were found. Detailed information on the dose of iodixanol, the period between iodixanol administration and anaphylactic reactions, and the clinical manifestations and outcomes of these reactions is available in cases described in case reports and is summarized in Table 1.

**Table 1:** Summary of reported cases of iodixanol-induced KS.

Reference	Age/Sex	Medical history	Dosage	Onset	KS Type	Clinical manifestations	Key treatment	Outcome
Present case	77//F	CHD, HTN, DM	50 ml	During infusion	II	Bradycardia, hypotension, convulsions	CPR, nitroglycerin, vasopressors, steroids, IABP	Recovery
[1] Zhiqiang, et al.	73/F	CHD, HTN, DM, breast cancer	50 ml	10 min	II	Rash, flushing, chest tightness	Dexamethasone, diphenhydramine, dopamine, nitroglycerin, and loratadine	Recovery (3 d)
[2] Kangzheng et al.	59/M	HTN, DM, AF	NR	During infusion	II	Rash, itching, chest tightness, nausea	Adrenaline	Recovery
[3] Dauvergne et al.	79/M	HTN, angina pectoris	NR	Immediate	II	Rash, erythema, chest pain	Steroids, antihistamines, salbutamol, CCB, nitrates, aspirin, statins	Recovery (3 d)

KS, Kounis syndrome; CHD, coronary heart disease; HTN, hypertension; DM, diabetes mellitus; AF, atrial fibrillation; CPR, cardiopulmonary resuscitation; IABP, intra-aortic balloon pump; CCB, calcium channel blocker; NR, not reported.

## Discussion

In this case report, we describe a 77-year-old Chinese woman with a diagnosis of CHD who was admitted to our hospital for further diagnosis and treatment. Coronary angiography was performed with the contrast agent iohexol 50 ml at a local hospital, and there was no allergic reaction to iohexol. However, coronary angiography with the contrast agent iodixanol was performed at our hospital, and the patient experienced fluctuations in blood pressure, initially rising to 215/85 mmHg and subsequently dropping to 60/40 mmHg. Similarly, the heart rate increased to 90 beats per min before progressively decreasing to 38 beats per min. Subsequently, it rose to 132 beats per min following standard emergency interventions, accompanied by symptoms of coma and convulsions. Angiography revealed significant spasm and stenosis in the left coronary artery. Symptoms were alleviated after emergency treatment; however, serum trypsin, a histamine metabolite (N-methylhistamine) in 24-h urine, and prostaglandin D2 (PGD2, 11-β-PGF2α) were undetectable. Therefore, on the basis of the diagnostic criteria (8), the diagnosis of “KS” may be considered.

The patient had no other medical history except for CHD and no history of drug or food allergies. Coronary angiography was performed at a local hospital, and no allergy to the iodine contrast agent occurred. No allergic adverse reactions occurred after long-term use of irbesartan, glimepiride, enteric-coated aspirin tablets or atorvastatin. The patient was not given any drugs other than iodixanol, and there was a definite relationship between the use of iodixanol injection and the occurrence of adverse reactions. Electrocardiography, color Doppler ultrasound and other examinations ruled out other pathological causes of KS. The patient received iodixanol as the contrast agent and was not on any concomitant medications. After the adverse reaction occurred, iodixanol was not used again, and no further episodes were observed. Both iodixanol instructions and published literature have documented this type of adverse reaction. Although a provocation test with iodixanol was not feasible, the patient had previously undergone a procedure with iohexol at a local hospital without any allergic reactions. It is hypothesized that the initial exposure to iohexol may have induced antibody production, leading to KS upon subsequent exposure to iodixanol. On the basis of the adverse drug reaction probability scale, the causal relationship between iodixanol and KS was categorized as “probable”.

KS is characterized by the simultaneous occurrence of acute coronary syndromes, such as coronary spasm, acute myocardial infarction, and stent thrombosis, in conjunction with conditions linked to mast-cell and platelet activation (9). The literature outlines four distinct types of KS (10). Type I denotes individuals with normal or nearly normal coronary arteries, where the release of inflammatory mediators can precipitate unstable angina or acute myocardial infarction, representing a subset of myocardial infarction with nonobstructive coronary arteries. In contrast, type II refers to patients with preexisting atheromatous disease whose clinical presentation may vary from inflammation-triggered unstable angina to acute myocardial infarction following plaque erosion or rupture. Additionally, patients classified as type III exhibit either stent thrombosis (subtype a) or stent restenosis (subtype b), characterized by the infiltration of inflammatory cells into the thrombus or coronary artery wall adjacent to the stent. Notably, KS can also manifest in patients with coronary grafts (type IV). The patient's underlying disease was CHD. During coronary angiography with iodixanol, the patient experienced fluctuations in heart rate and blood pressure, along with unconsciousness and convulsive symptoms. Angiography revealed severe spasm and stenosis of the left coronary artery, which was consistent with type II KS.

Risk factors for KS include a history of allergies, hypertension, diabetes, smoking and hyperlipidemia. Approximately 25% of patients with KS have a known history of allergies (3). KS can occur at any age, but it is most commonly observed in individuals between the ages of 40 and 70 years (3).

The mechanism of KS can be initiated by an immunoglobulin E (IgE)-mediated hypersensitivity reaction, where IgE attaches to high-affinity receptors (FcεRIs) on mast cells, sensitizing them. Upon re-exposure to an allergen, mast cell degranulation occurs, leading to the release of diverse mediators that play a role in the development of KS (11). The pathophysiology of contrast-related hypersensitivity reactions may share features with endothelial dysfunction in patients with mastocytosis. The latter is a multifactorial process driven primarily by the combined effects of massive mast cell mediator release (e.g., tryptase, histamine, PDG₂), direct mast cell infiltration into the vasculature and subsequent oxidative stress. Importantly, the severity of endothelial dysfunction is closely correlated with the systemic mast cell burden, as indicated by serum tryptase levels (12). The pathophysiological mechanism of iodixanol-induced KS primarily involves type I hypersensitivity and mast cell activation. Iodixanol triggers mast cell activation via both IgE-dependent and non-IgE-dependent pathways (e.g., complement, MRGPRX2), leading to the release of various vasoactive mediators, including histamine, tryptase, chymase, matrix metalloproteinases (MMPs), chymase, leukotrienes (LTC4, LTD4, LTE4), prostaglandin D2 (PGD2), and thromboxane A2 (TxA2), as well as inflammatory factors such as tumor necrosis factor-alpha (TNF-α), interleukin-6 (IL-6), granulocyte‒macrophage colony-stimulating factor (GM-CSF), chemokines (CCL2/3/5), and platelet-activating factor (PAF). These mediators collectively induce coronary artery spasm and promote plaque instability or thrombosis, ultimately culminating in the manifestation of KS (11).

KS is not a rare disease, but it is frequently underdiagnosed in clinical settings and can easily be overlooked. In the USA, an annual prevalence of 1.1% for KS has been estimated on the basis of an analysis of 253,420 patients who experienced allergic reactions over seven years (13, 14). The mortality of KS is 2.9% (3). It is important to consider this disease when evaluating patients with chest pain or suspected myocardial infarction, particularly if allergic reaction symptoms are present. Close attention should be given to the time interval between exposure to the trigger and the onset of symptoms (3). Although most patients show symptoms within an hour of exposure, nearly 10% have been reported to develop symptoms more than six hours after exposure (14). The diagnosis of KS was based on clinical presentation; a history of previous allergies; laboratory tests, such as troponin Ⅰ, eosinophil count, serum trypsin, histamine metabolite (N-methylhistamine) in 24-h urine, and prostaglandin D2 (PGD2, 11-β-PGF2α) and the levels of cardiac enzymes (CK, CK-MB), C-reactive protein, immunoglobulin E and interleukin-6; and findings from ECG, such as ST elevation myocardial infarction; and coronary angiography, such as coronary vasospasm, echocardiography and magnetic resonance imaging (3, 8, 15). The management of KS requires an integrated approach that simultaneously addresses acute coronary syndrome (ACS) and the underlying anaphylactic reaction, in accordance with their respective evidence-based guidelines (16, 17). The treatment of KS should manage acute coronary syndrome and severe allergic reactions simultaneously, tailored on the basis of the initial presentation, such as vasospasm, thrombosis or plaque rupture, that results from immune reactions (3, 15). Notably, anaphylactic shock with peripheral vasodilation necessitates vasopressors, whereas coronary vasospasm requires vasodilators (4).

Additionally, certain drugs used to treat cardiac manifestations may exacerbate allergic reactions, and vice versa, further complicating treatment, such as adrenaline, nitrates, and aspirin (18, 19). To minimize therapeutic conflicts, the intracoronary use of nitroglycerin is recommended. If a patient is taking β-blockers, glucagon should be administered, as β-blockers can induce resistance to adrenaline. Notably, most anaphylactic reactions respond to initial treatment with a single dose of adrenaline; approximately 10% require two doses, and approximately 2% require more than two doses (14). Furthermore, in the type III variant, aspiration of the intrastent thrombus should be performed. Physicians should ask patients and their family members about previous drug allergies, particularly allergies to iodine contrast agents, before prescribing iodixanol. If there is a history of iodine contrast agent allergy, iodixanol should be avoided. Iodine skin allergy tests can be conducted before patients are prescribed iodixanol. During the use of iodixanol, the patient's skin and ECG data, including blood pressure and complaints, should be carefully monitored to allow the early diagnosis of KS and prompt discontinuation of iodixanol therapy. Once KS is diagnosed, prompt therapy should be initiated.

We used IABP for KS in our patient. IABP is the most commonly used mechanical circulatory support device and has been used to improve hemodynamic parameters in patients with cardiogenic shock for more than four decades (20). It can help reduce afterload, enhance cardiac output, improve coronary blood flow, and decrease oxygen consumption (21). The IABP balloon rapidly inflates during the heart's diastolic phase, leading to a rise in aortic diastolic pressure, which can increase by 30%–70% compared with baseline levels. Prior to the systolic phase, the balloon rapidly deflates, causing a 5%–30% reduction in aortic pressure (end-diastolic aortic pressure), which decreases left ventricular afterload and increases cardiac output by 0.5–1.0 L/min, with a maximum increase of approximately 15%. These effects significantly enhance the balance between myocardial oxygen supply and demand and improve coronary blood flow beneath the endocardium (22). Therefore, this case also highlights the successful use of the IABP to stabilize hemodynamic parameters after the failure of conventional emergency treatments. To the best of our knowledge, this is the first reported case of IABP use in iodixanol-induced KS.

## Conclusion

This report presents the first use of IABP to reverse life-threatening, refractory hypotension in iodixanol-induced KS patients. This finding underscores a key clinical lesson that when standard therapy fails to stabilize a patient with severe allergic coronary vasospasm, the IABP provides essential hemodynamic support. This case highlights the need to recognize KS and be ready to escalate to mechanical circulatory support in treatment-resistant situations.

## Data Availability

The original contributions presented in the study are included in the article/Supplementary Material, further inquiries can be directed to the corresponding authors.
